# Naringenin Induces HepG2 Cell Apoptosis via ROS-Mediated JAK-2/STAT-3 Signaling Pathways

**DOI:** 10.3390/molecules28114506

**Published:** 2023-06-01

**Authors:** Ming Zhang, Jianmei Lai, Qianlong Wu, Jia Lai, Jingyao Su, Bing Zhu, Yinghua Li

**Affiliations:** 1 Department of Interventional Radiology and Vascular Anomalies, Guangzhou Women and Children’s Medical Center, Guangzhou Medical University, Guangzhou 510120, China; 2023760758@gzhmu.edu.cn; 2 Center Laboratory, Guangzhou Women and Children’s Medical Center, Guangzhou Medical University, Guangzhou 510120, China

**Keywords:** naringenin, cancer, ROS, caspase-3, apoptosis

## Abstract

Hepatocarcinoma is one of the most prevalent digestive system tumors worldwide and lacks effective therapy. Recently, naringenin has been isolated from some citrus fruits, and its anticancer effects have been tested. However, the molecular mechanisms of naringenin and the potential implications of oxidative stress in naringenin-induced cytotoxicity in HepG2 cells remain elusive. Based on the above, the present study examined the effect of naringenin on the cytotoxic and anticancer mechanisms of HepG2 cells. Naringenin-induced HepG2 cell apoptosis was confirmed via the accumulation of the sub-G1 cell population, phosphatidylserine exposure, mitochondrial transmembrane potential loss, DNA fragmentation, caspase-3 activation, and caspase-9 activation. Furthermore, naringenin enhanced cytotoxic effects on HepG2 cells and triggered intracellular reactive oxygen species; the signaling pathways of JAK-2/STAT-3 were inhibited, and caspase-3 was activated to advance cell apoptosis. These results suggest that naringenin plays an important role in inducing apoptosis in HepG2 cells and that naringenin may be a promising candidate for cancer therapy.

## 1. Introduction

Liver cancer, ranked as the third leading cause of cancer-related mortality by the World Health Organization (WHO) and fifth in terms of incident cases globally [1], poses a significant health burden. The WHO also estimates that more than 1 million individuals will die from liver cancers in 2030 [2]. Among the various types, hepatocellular carcinoma (HCC) accounts for approximately 90% of primary liver cancer cases [3]. The pathophysiological mechanism of HCC is complex, which is usually caused by malignant transformation of hepatocytes. There are many risk factors for HCC, mainly including hepatitis B and C virus, aflatoxin B1, excessive alcohol consumption, smoking, diabetes, and obesity [4]. The majority of HCC patients are frequently diagnosed in an advanced stage with a poor prognosis due to the lack of particular symptoms in early stages, the lack of early diagnostic markers, and the low percentage of radical resectable HCC at diagnosis [5]. According to the SEER database of the National Cancer Institute, the average 5-year survival rate for HCC patients is 19.6%, but patients with advanced, metastatic disease may have a 5-year survival rate as low as 2.5% [6]. Hepatocellular carcinoma is a complex disease that often develops from cirrhosis and requires a multidisciplinary approach involving diagnosis, staging, therapy, and monitoring in specialist outpatient clinics to minimize disease progression. Although the clinical treatment for hepatocellular carcinoma has significantly advanced in the last decade, the recurrence rate of this disease after ablation or surgical resection is high, and there is also a lack of effective chemotherapy for cirrhotic patients and adjuvant therapy for cancer patients after surgery [7]. Therefore, delivering effective treatment strategies is one of the main challenges for HCC.

As part of the treatment strategy for HCC, surgical resection, liver transplantation, percutaneous local ablation, transarterial embolization, chemotherapy, and radiotherapy are available options. Moreover, it is crucial for cancer therapy to kill cancer cells without damaging normal cells, which is especially important for patients with advanced cancer. Hepatocellular cells can evade immune detection in a series of complex ways, thereby avoiding programmed death [8]. Therefore, inducing apoptosis is a major objective of anticancer therapies. Recently, it was shown that several natural compounds have the ability to kill cancer cells by causing apoptosis. Additionally, these medications may be used with radiation or chemotherapy, which can enhance the therapeutic efficacy and reduce the side effects of many common cancer treatments [9,10]. Consequently, natural products are considered potential candidates due to their low side effects and high anticancer activity [11].

Modern pharmaceuticals rarely provide relief for hepatic diseases, leading to the frequent utilization of plant-based treatments in liver disorder management. Although there are currently few drugs available to treat liver diseases, herbal remedies have grown in importance and popularity in recent years due to their potency, purity, and accessibility [12]. Natural substances derived from healing plants or fruits, as well as complementary and alternative medicine, have recently demonstrated the potential activity to stop the formation of tumor cells and lessen inflammation in cancer cells [13]. Flavonoids are a wide-ranging class of phytonutrients present in almost all kinds of vegetables and plants [14]. Researchers have found that flavonoids have a wide range of beneficial effects, including antioxidant, antiviral, antibacterial, and antiallergenic effects [15,16]. Naringenin (Nar) is a flavanone and aglycone of naringin with diverse pharmacological activities, predominantly found in citrus fruits [17]. In vitro and in vivo studies of naringenin have revealed its antioxidant, anti-inflammatory, antidiabetic, and anticancer properties, and naringenin can also improve the central nervous system and be used to treat cardiovascular diseases. Otherwise, naringenin has been demonstrated to suppress tumor growth by inhibiting cell proliferation and stimulating apoptosis, including in breast, bladder, and cervical cancer [18]. Conventional antiangiogenic therapies have limitations and may even accelerate cancer progression. However, at the advanced stages of carcinogenesis, a number of gene products, particularly tumor inhibitors, can function as the specific targets for naringenin to halt the growth of cancer. A meta-analysis revealed that the intrinsic apoptotic proteins caspase-3 and 9 were both considerably increased in a variety of tumor cells after naringenin treatment [13]. These results emphasize the naringenin’s potential therapeutic value in the treatment of cancer. Studies on the antioxidant effect of naringenin can be considered ambiguous, and it may depend on the formation of radicals, the model used, and the flavonoid concentration [19]. Thus far, the antioxidant effect of naringenin has been widely applied in the field of medicine, but its pro-oxidative mechanism in tumor cells is not yet clear. Under certain experimental conditions, the molecular mechanism of naringenin increases the level of ROS in HepG2 cells, and the initiation of its apoptosis pathway remains to be further studied. Therefore, through exploring the molecular mechanism of naringenin inducing oxidative damage-related apoptosis in HepG2 cells, this study provides a theoretical basis for the development and application of therapeutic drugs for liver cancer.

## 2. Results and Discussion

### 2.1. In Vitro Cytotoxicity of Naringenin

**Figure 1:**
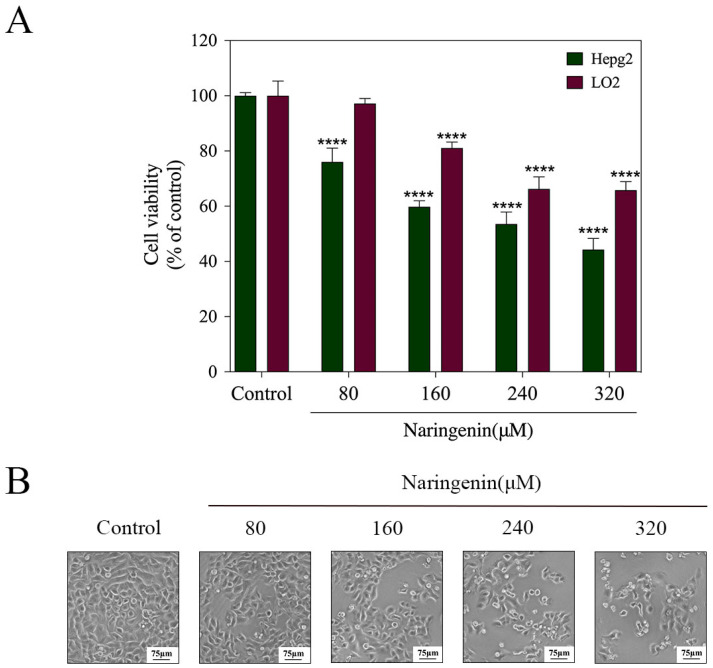
Effects of different concentrations of naringenin on the growth of HepG2 and LO2 cells. (**A**) Cell viability was evaluated after treatment with different concentrations of naringenin for 24 h using the Cell Counting Kit-8 assay. (**B**) Morphological changes in HepG2 cells with different concentrations of naringenin. Bars with different characters are statistically different at **** *p* < 0.0001.

The antitumor activity of naringenin at varying concentrations was assessed through the utilization of the CCK-8 method, enabling the determination of cell viability and evaluation of its growth status. Figure 1A shows that naringenin exhibited a significantly less inhibitory effect on LO2 normal liver cells compared with HepG2 cells, and these inhibitory effects were dose-dependent under the action of 80 μM (76.03% vs. 94.97%), 160 μM (59.77% vs. 81.04%), 240 μM (53.02% vs. 66.25%), and 320 μM of naringenin (44.27% vs. 64.79%). As shown in Figure 1B, the effects of different concentrations of naringenin on the inhibition of HepG2 cell proliferation were further confirmed. Following treatment with varying concentrations of naringenin, a decrease in cell count was observed, accompanied by cellular shedding, loss of adhesion, cytoplasmic shrinkage, and a transition to a rounded morphology. In summary, all tested concentrations of naringenin within the range of 80–320 μM exhibited inhibitory effects on the growth of HepG2 cells, with the most significant inhibitory effect observed at higher concentrations.

### 2.2. Depletion of Mitochondrial Membrane Potential (ΔΨm) Induced by Naringenin

**Figure 2:**
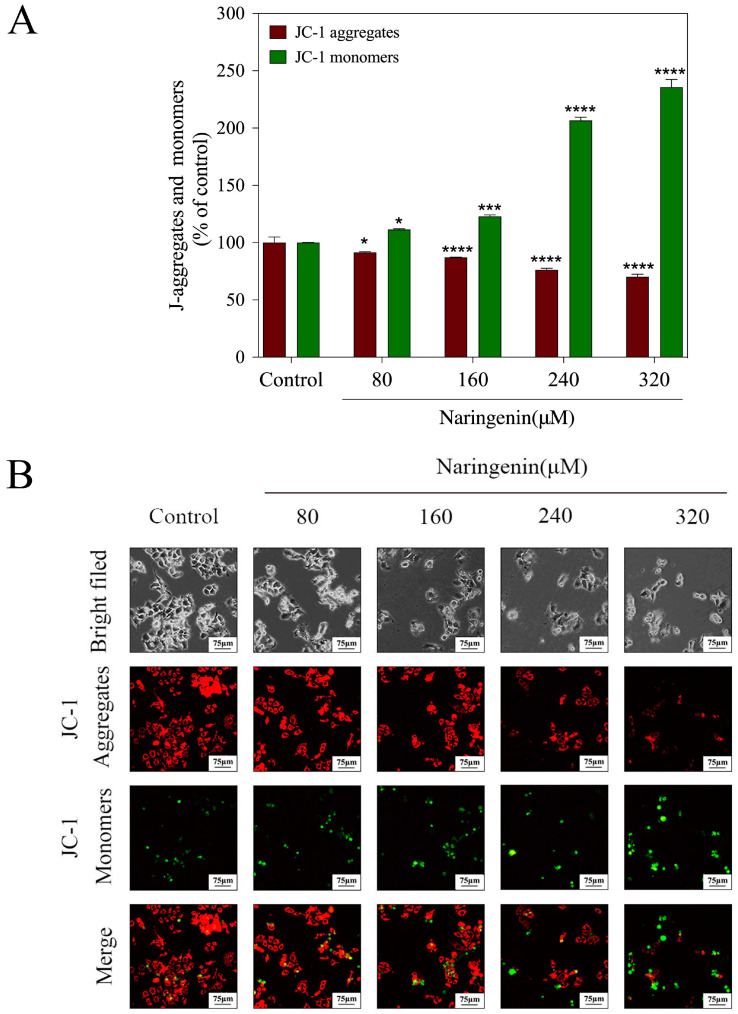
Depletion of mitochondrial membrane potential induced with different concentrations of naringenin. (**A**) The percentage of J-aggregate and monomers. (**B**) JC-1 aggregates and monomers of HepG2 cells were exposed to different concentrations of naringenin under an immunofluorescence microscope. Bars with different characters are statistically different at * *p* < 0.05, *** *p* < 0.001, and **** *p* < 0.0001.

The depletion of mitochondrial membrane potential (ΔΨm) is a critical event in the early stages of apoptosis [20]. The mitochondrial depolarization of HepG2 cells treated with naringenin was enhanced, and the functions of cells were disordered. Treatment of HepG2 cells with naringenin resulted in an enhanced mitochondrial depolarization, leading to disrupted cellular functions. As depicted in Figure 2A, the red fluorescence values significantly decreased in the naringenin treatment groups compared to those in the control group: 91.45% (80 μM), 87.17% (160 μM), 76.20% (240 μM), and 70.12% (320 μM). Conversely, the green fluorescence values in the naringenin treatment groups significantly increased compared to those in the control group: 111.42% (80 μM), 122.95% (160 μM), 206.68% (320 μM), and 235.74% (320 μM). As shown in Figure 2B, JC-1 was present in the form of polymers in the mitochondria of Hepg2 cells in the control group, showing a bright red fluorescence, whereas the green fluorescence in cells was very weak. After treatment with different concentrations of naringenin, the mitochondrial membrane potential gradually decreased with the increase in concentration, JC-1 was not present in the mitochondrial matrix in the form of a polymer, and the intensity of the red fluorescence in mitochondria gradually decreased, while the green fluorescence in the cytoplasm gradually increased. It can be concluded that the mitochondrial depolarization of HepG2 cells treated with naringenin was enhanced, and their function was disturbed. 

### 2.3. Translocation of Phosphatidylserine Induced by Naringenin

**Figure 3:**
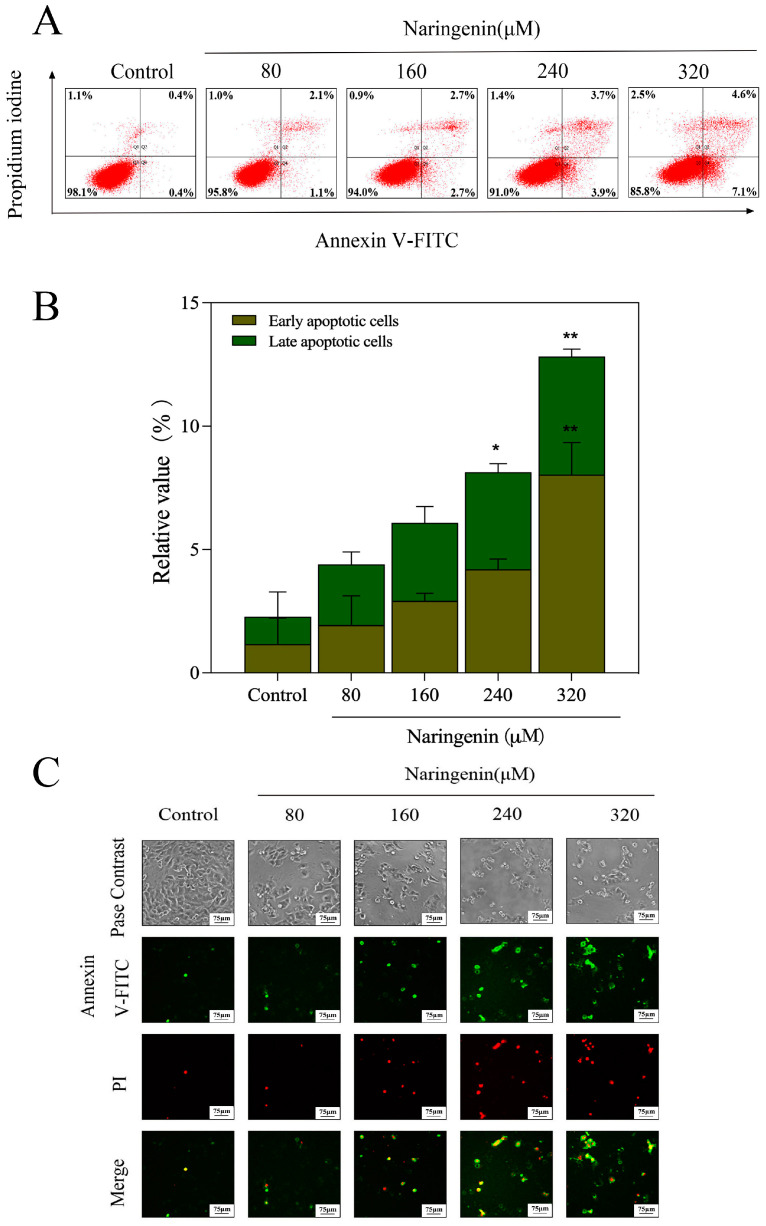

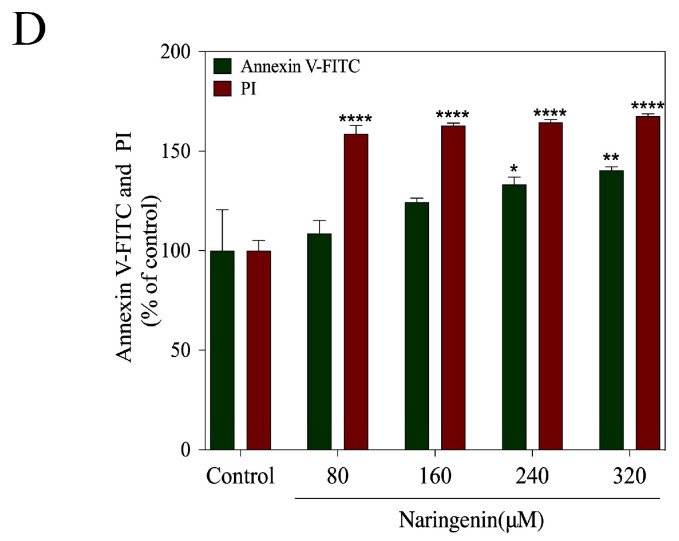
Translocation of phosphatidylserine induced by different concentrations of naringenin in HepG2 cells. (**A**) The upper right quadrant represents the cells in the late stage of apoptosis or necrosis, and the lower right represents cells in the early stage of apoptosis. (**B**) Apoptotic cell histogram. (**C**) Annexin V-FITC and PI of HepG2 cells were exposed to different concentrations of naringenin under an immunofluorescence microscope. (**D**) The mean fluorescence intensity of FITC and PI. Bars with different characters are statistically different at * *p* < 0.05, ** *p* < 0.01, **** *p* < 0.0001.

The apoptosis of HepG2 cells treated with different naringenin concentrations (80, 160, 240, and 360 μM) was detected with flow cytometry using the Annexin-FITC/PI double-labeling method. Flow cytometry (Figure 3A,B) revealed that HepG2 cells induced significant apoptosis after 24 h treatment with naringenin. With the increase in naringenin concentration, the cell apoptosis rate increased from 0.4% to 7.1%. As shown in Figure 3C,D, compared with that in the control group, the intensity of red and green fluorescence gradually increased, from 108.68% to 140.41% and from 158.67% to 167.59%, respectively, with the increase in the concentration of naringenin. This indicated that the number of early apoptotic cells and late apoptotic cells increased. These results suggest that the proliferation inhibition of HepG2 cells may be associated with apoptosis.

### 2.4. Naringenin-Induced HepG2 Cell Apoptosis

**Figure 4:**
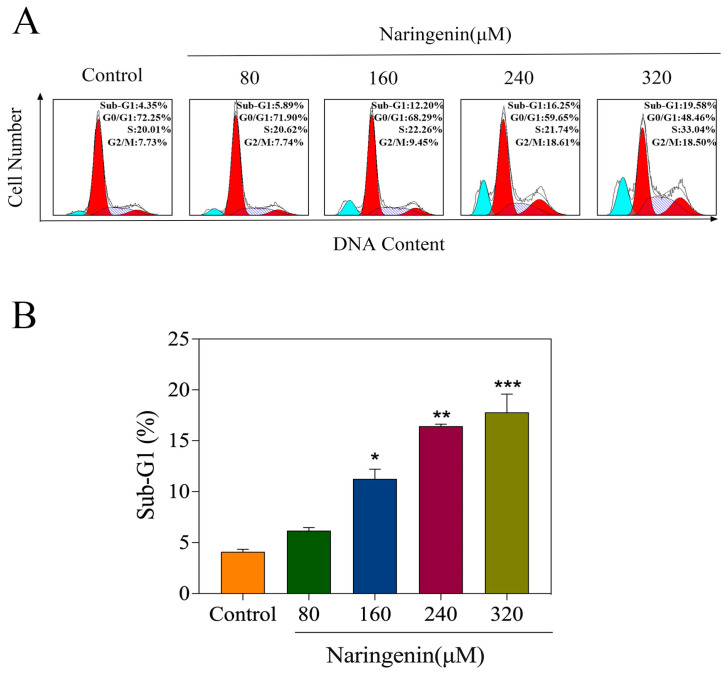
Naringenin-induced apoptosis in HepG2 cells. (**A**) The cell cycle distribution with different concentrations of naringenin was analyzed by quantifying DNA content via flow cytometric analysis. (**B**) Histogram of sub-G1 peak. Bars with different characters are statistically different at * *p* < 0.05, ** *p* < 0.01, and *** *p* < 0.001.

The peak of apoptotic cells was represented by the Sub-G1 peak on the fluorescence map of the flow cytometer. To determine if naringenin-induced cell death was connected to apoptosis, this study employed PI-flow cytometry. Figure 4A,B show that there were noticeably more apoptotic cells below G1 in the naringenin DNA histogram in comparison to the control group. For instance, the number of apoptotic cells dramatically increased from 5.89% to 19.58% in HepG2 cells treated with various dosages of naringenin, with no discernible change in the G0/G1, S, or G2/M phases. As shown in Figure 4B, starting from the naringenin concentration of 160 μM, the sub-G1 apoptotic cell population significantly increased. The findings above demonstrate that naringenin affects cell apoptosis.

### 2.5. Detection of Caspase-3 Activity

**Figure 5:**
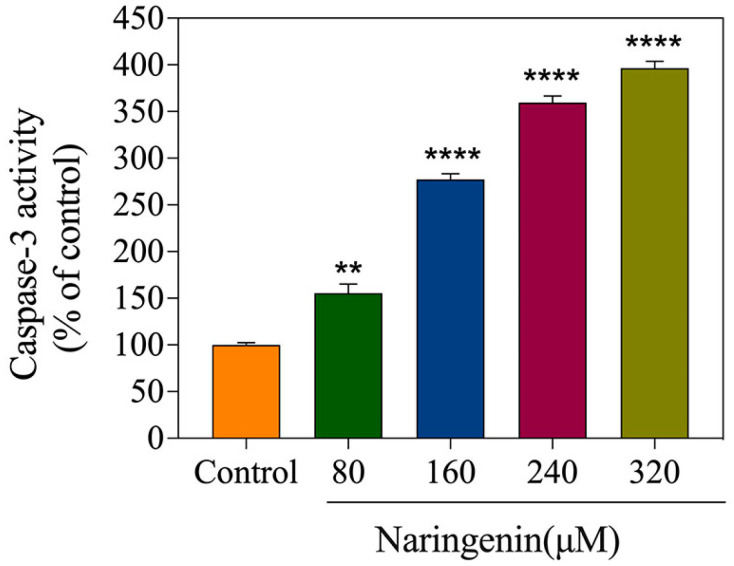
The activation of caspase-3 triggered by different concentrations of naringenin examined via synthetic fluorogenic substrates. Bars with different characters are statistically different at ** *p* < 0.01 or **** *p* < 0.0001.

Caspase-3 activation allowed apoptosis to be identified, and naringenin was used to treat HepG2 cells at various doses. Caspase-3 activity was detected using an enzyme labeling device. As shown in Figure 5, the caspase-3 activity of the control cells without treatment was arbitrarily expressed as 100%. Compared with that in the control group, the caspase-3 activity in the naringenin treatment group significantly increased, with percentages of 155.51% (80 μM), 277.36% (160 μM), 359.63% (240 μM), and 396.46% (360 μM), respectively. The results suggest that naringenin may induce apoptosis via caspase-3.

### 2.6. Detection of Caspase-9 Activity

**Figure 6:**
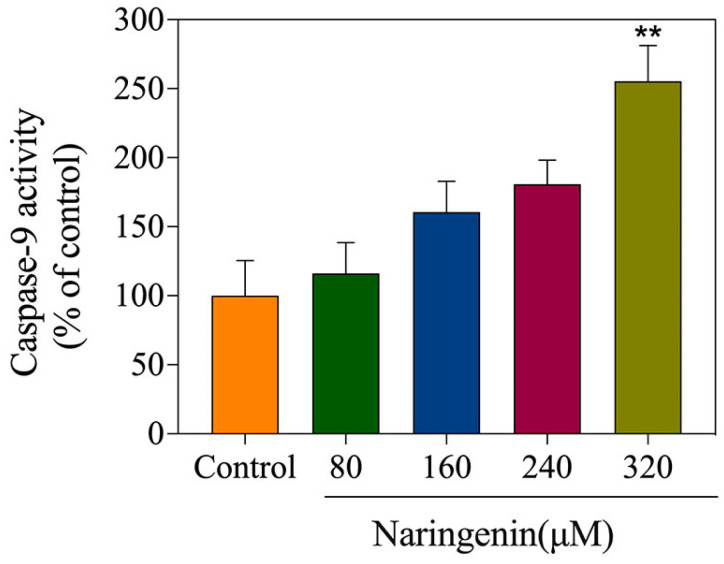
The activation of caspase-9 triggered by different concentrations of naringenin examined via synthetic fluorogenic substrates. Bars with different characters are statistically different at ** *p* < 0.01.

Caspase-9 can efficiently cleave intracellular proteins and participate in the regulation of cell apoptosis. The caspase-9 activity was detected with a caspase-9 activity assay kit [21]. As shown in Figure 6, the caspase-9 activity of the control cells without treatment was arbitrarily expressed as 100%. In the naringenin treatment group, the caspase-9 activity was higher than in the control group, with percentages of 116.17% (80 μM), 160.59% (160 μM), 180.75% (240 μM), and 255.35% (360 μM), respectively. In summary, naringenin may induce apoptosis through caspase-9.

### 2.7. Induction of ROS Generation by Naringenin

**Figure 7:**
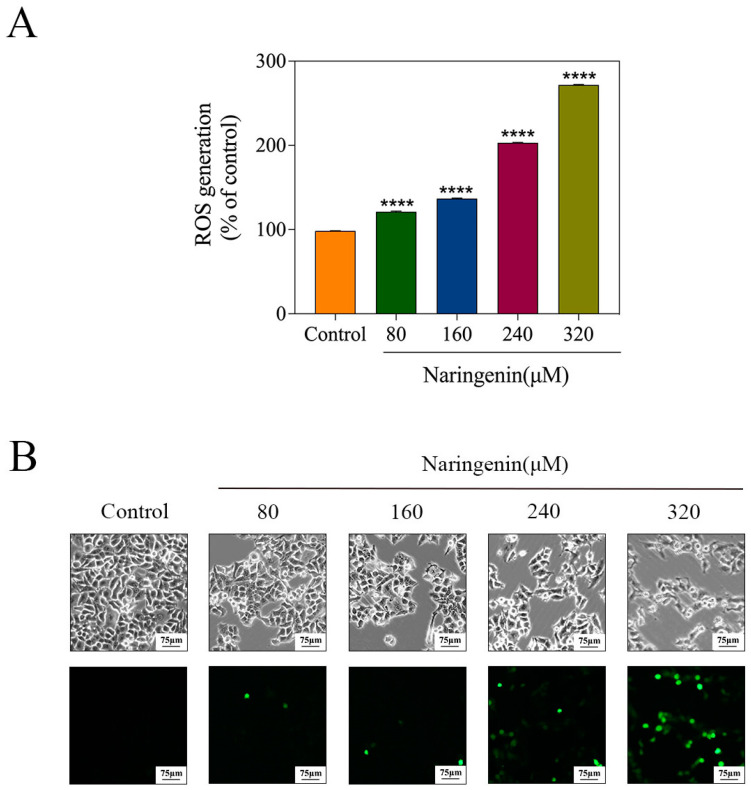
ROS overproduction induced by naringenin of HepG2 cells. (**A**) Changes in intracellular ROS generation detected by measuring DCF fluorescence intensity. (**B**) HepG2 cells were incubated with ten µM of DCF-DA in a serum-free medium for 20 min and then treated with different concentrations of naringenin under a phase-contrast microscope. Bars with different characters are statistically different at **** *p* < 0.0001.

As a result of intracellular ROS, nonfluorescent DCFH can be turned into fluorescent DCF through oxidation. By analyzing DCF fluorescence intensity levels, it was possible to determine whether intracellular ROS are produced [22]. DCF-DA was used to detect ROS production to reveal whether naringenin induced ROS production. After treatment with different concentrations of naringenin, intracellular ROS production increased from 121.18% to 271.74%, as shown in Figure 7A. As shown in Figure 7B, naringenin detects the fluorescence intensity of DCF. The phosphor of the naringenin treatment group was more effective than that of the control group. These results show that naringenin can effectively promote the production of ROS.

### 2.8. Activation of ROS-Mediated Signaling Pathways by Naringenin

**Figure 8:**
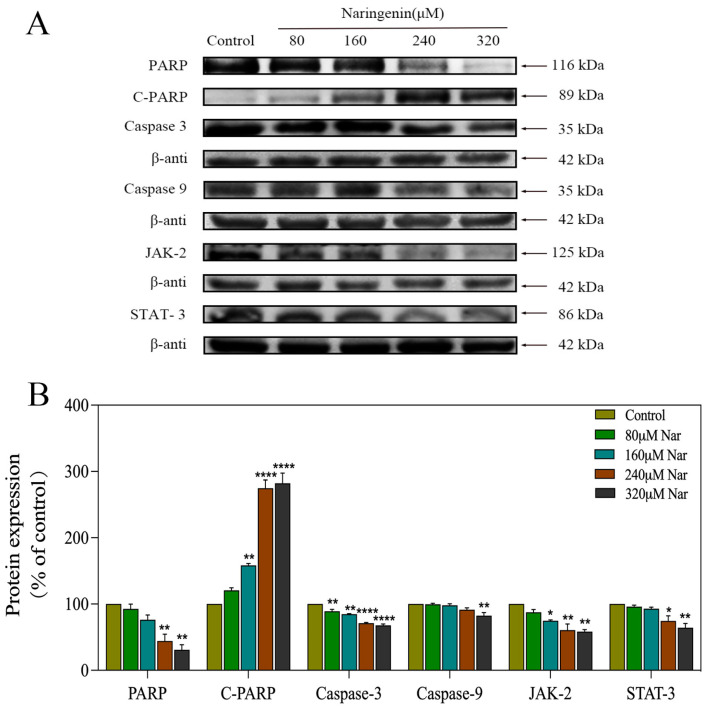
Activation of intracellular apoptotic signaling pathways by naringenin in HepG2 cells. (**A**) The proapoptotic and antiapoptotic proteins expressed by HepG2 cells exposed to different concentrations of quercetin were tracked with Western blotting. (**B**) Bands were quantified, and results are expressed as a percentage of control; β-actin was used as the loading control. Bars with different characters are statistically different at * *p* < 0.05, ** *p* < 0.01 and **** *p* < 0.0001.

**Figure 9:**
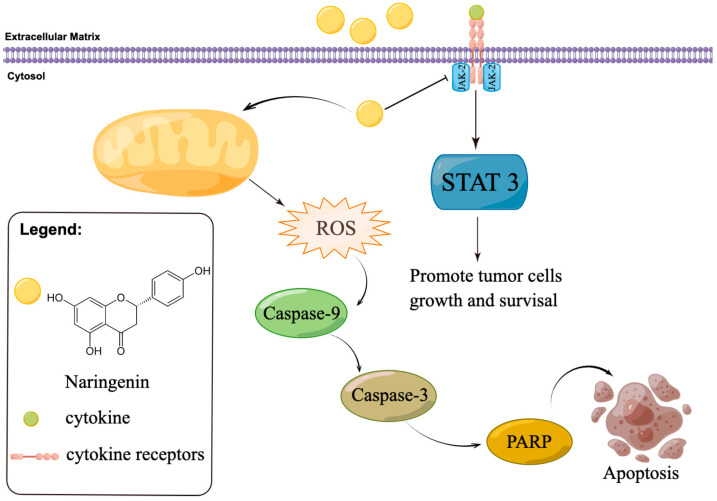
Apoptotic signaling pathways regulated by naringenin in HepG2 cells. The apoptotic signaling pathways were induced by naringenin via ROS-mediated JAK-2/STAT-3 and caspase-3 signaling pathways.

Western blotting was used to identify apoptosis-related proteins and to assess their involvement in and contribution to apoptosis. As seen in Figure 8A,B, caspase-3 and PARP expression levels were significantly downregulated following naringenin therapy. Additionally, naringenin increased the level of cleaved PARP expression. The outcomes demonstrate that naringenin dramatically increased caspase-3 activation and downstream PARP cleavage (Figure 8A,B). In addition, activated caspase-9 was found to activate the apoptotic executive protein caspase-3 to promote subsequent apoptotic signaling. For the caspase-9 signal pathway, the expression of caspase-9 was downregulated after naringenin treatment (Figure 8A,B). As shown in Figure 8A,B, HepG2 cells treated with naringenin could effectively reduce the expression of JAK-2 and STAT-3 in HepG2 cells. These results suggest that naringenin inhibits the growth and proliferation of HepG2 cells by inhibiting the JAK-2/STAT-3 pathway. In summary, naringenin induced apoptosis in HepG2 cells by regulating ROS-mediated JAK-2/STAT-3 and caspase-3 signaling pathways (Figure 9).

## 3. Discussion

**Figure 10:**
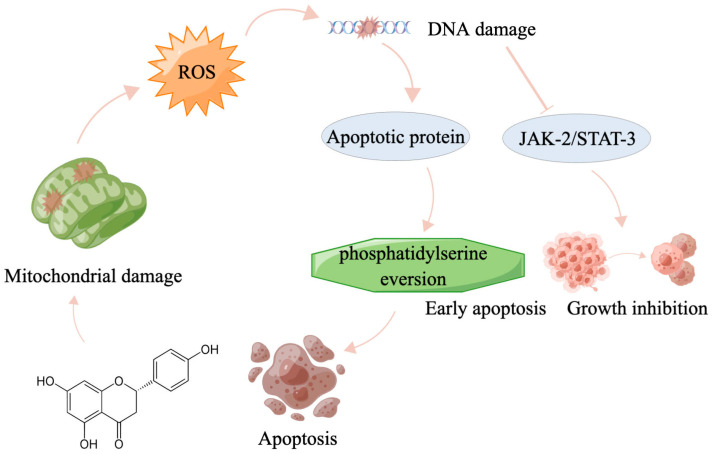
Naringenin promotes the production of ROS by regulating mitochondrial damage and causing DNA damage, thereby activating the apoptosis pathway and limiting the role of the JAK-2/STAT-3 signaling pathway.

Naringenin is an important phytochemical belonging to the flavonoid group of polyphenols and is mainly found in citrus fruits such as grapefruit. It has the potential to treat different types of cancer. Naringenin can inhibit cancer progression via multiple mechanisms, such as apoptosis induction, cell cycle arrest, mitochondrial damage, ROS accumulation, and the modification of various signaling pathways [18]. This study evaluated the antitumor suppressive activity of naringenin on HepG2 cells (Figure 10).

CCK-8 contains WST-8, which can be reduced by dehydrogenase in mitochondria, and the detection value can indicate the cell activity [23]. Consequently, the color has a linear correlation with the number of HepG2 cells. By using a microplate spectrophotometer, we found that naringenin had a strong cytotoxic effect on HepG2 cells and a low cytotoxic effect on normal liver cell LO2, indicating the selective cytotoxicity of naringenin. These findings indicate that naringenin effectively inhibits HepG2 cell vitality, induces its breakdown, and is more destructive to HepG2 cells at the same concentration, potentially reducing side effects in clinical applications. 

When the ΔΨm was high, JC-1 aggregated in the mitochondrial matrix and formed polymers (JC-1-aggregates), which could produce red fluorescence; otherwise, it would produce green fluorescence. A decrease in mitochondrial membrane potential can be detected via the transition of JC-1 from red to green fluorescence, which can also be used as an apoptosis detection indicator [20]. Our study indicates that naringenin significantly decreased mitochondrial membrane potential, thus promoting apoptosis in HepG2 cells. It can be concluded that naringenin induces apoptosis by decreasing mitochondrial dysfunction in HepG2 cells.

Adjacent to the cytoplasm, phosphatidylserine is primarily found in the inner leaflet of the plasma membrane. In the early stages of apoptosis, different cell types turn phosphatidyl serine to the cell surface or the outside of the cell membrane. Annexin-V-FITC can be bound to phosphatidylserine and used to detect the eversion of phosphatidylserine. Additionally, propidium iodide can produce a red fluorescence in necrotic cells or cells that lose the integrity of their cell membrane at the late stage of apoptosis. Since the integrity of the cell membrane is compromised in necrotic cells, Annexin V-FITC can enter the cytoplasm and mix with the phosphatidylserine present on the inner side of the cell membrane to produce green fluorescence in necrotic cells. The apoptotic cells induced by naringenin were detected using Annexin-V/PI double staining [24]. As shown in Figure 3A, the dot pattern of the HepG2 cell treatment group showed that its numbers of Annexin V-FITC-positive and PI-negative cells, namely early apoptotic cells, significantly increased with the increase in concentration. Annexin V-FITC, PI-stained double-positive cells (late apoptotic cells), and necrotic cells also increased in concentration (Figure 3A,B). As shown in Figure 3C, the number of apoptotic cells and Annexin V-FITC/PI in naringenin-treated HepG2 cells increased by comparison. The results show that naringenin inhibits the proliferation of HepG2 cells mainly via apoptosis. 

Propidium (PI) is a fluorescent dye for double-stranded DNA. The combination of propidium iodide and double-stranded DNA can produce fluorescence, and the fluorescence intensity is proportional to the content of double-stranded DNA. The DNA content of cells was determined via flow cytometry following staining with DNA dye propidium iodide (PI). DNA fragments are created when DNA breaks in apoptotic cells between nucleosomes. As a result, DNA fragmentation is a crucial indicator for determining apoptosis. Due to the condensation of the nucleus and the DNA fragmentation of the apoptotic cells, some of the genomic DNA fragments were lost during the staining process. Therefore, the apoptotic cells showed significantly weak staining after propidium iodide staining, i.e., the fluorescence intensity decreased. Then, the distribution of DNA content was judged according to fluorescence intensity via an analysis of cell cycle and apoptosis. The experimental results showed that naringenin can cause DNA damage in cells, and the higher the concentration is, the more DNA fragments are produced. DNA fragmentation is an important biochemical hallmark of cell apoptosis. The induction of apoptosis was further confirmed using a DAPI costaining assay.

A large proportion of reactive oxygen species (ROS) formed in cells is derived from the electron transport chain in mitochondria during cellular respiration [25]. It has also been found that p66Shc is an oxidoreductase, directly stimulating mitochondrial ROS generation. The mitochondrial membrane forms ROS when p66Shc oxidizes cytochrome c (Cyt-c) [26]. In general, excess cellular ROS accumulation is shown to have negative effects since ROS are potential carcinogens because of their role in the regulation of tumor progression [27,28]. The substance dichlorodihydrofluorescein has no fluorescence of its own and can freely penetrate the membrane of the cell and be hydrolyzed by esterase into dichlorodihydrofluorescein (DCFH) [29]. DCFH, on the other hand, cannot penetrate the cell membrane, allowing the probe to be easily loaded into the cell. The amount of DCFH in HepG2 cells treated with naringenin increased, indicating that naringenin can promote the accumulation of ROS in HepG2 cells, thereby initiating the subsequent apoptotic pathway.

Importantly, cysteine proteinases (caspases) play a significant role in signaling pathways that lead to apoptosis in cells. Caspase-3, also known as CPP32, Yama, or apopain, belongs to the CED-3 subfamily (CED-3 subfamily) of the caspase family, and sequential cleavage of caspase-3 as its activation plays a dominant role in the execution phase of cell apoptosis. In addition to cleaving procaspase 2, 6, 7, and 9, caspase-3 may also precisely and directly cleave a wide variety of caspase substrates, such as PARP (poly (ADP-ribose) polymerase), ICAD (inhibitor of caspase-activated deoxyribonuclease), gelsolin, and fodrin. The molecular process of apoptosis includes this crucial caspase-3-mediated protein splicing [30]. Caspase-3 is a major executor of apoptosis, cleaving many functional proteins. Caspase-9 is an upstream protease in the apoptotic signal transduction process [31]. Activated caspase-9 can activate caspase-3, the most critical enzyme of apoptosis, thus promoting a subsequent apoptosis signal. The excessive production of intracellular ROS can lead to DNA damage and a series of different signal pathways, such as the JAK-2/STAT-3 and caspase-9 signal pathways. Since excessive production of ROS was detected in cells exposed to naringenin, we used Western blotting to determine the role of ROS-mediated signaling pathways. It was found that naringenin can not only activate the caspase-9/caspase-3/PARP/C-PARP apoptosis pathway but also inhibit tumor cell proliferation and growth via the inhibition of JAK-2/STAT-3.

Accumulating evidence suggests that activation of the JAK2-STAT3 signaling pathway by growth factors or cytokines plays an active role in tumor growth and progression [32]. It has also been demonstrated that the JAK2-STAT3 signaling pathway plays an important role in inflammation response and liver cancer progression [33]. After cytokines activate JAK-2, STAT-3 protein phosphorylates, dimerizes, transfers to nucleic acid, and begins gene transcription [22]. STAT3 has been reported to be activated in many cancers, and STAT3 signaling is considered an important process in malignant transformation [34] and angiogenesis induction [35]. However, our study found that naringenin can significantly downregulate the expression of JAK-2 and STAT-3, which indicates that naringenin can limit the activity of HepG2 cells to a certain extent via JAK-2/STAT-3.

## 4. Materials and Methods

### 4.1. Materials

HepG2 cells were obtained from the American Type Culture Collection (ATCC^®^, CCL-13TM). LO2 cells (regular human liver cells line) were provided by the Cell Bank of the Chinese Academy of Sciences (Shanghai, China). They were cultured with serum-free minimum essential medium (MEM; Gibco, Grand Island, NY, USA) at 37 °C. DAPI and DCF-DA were purchased from Sigma-Aldrich. Dulbecco’s Modified Eagle’s Medium (DMEM) and fetal bovine serum (FBS) were purchased from Gibco (Life Technologies, Carlsbad, CA, USA). Caspase-3, poly (ADP-ribose) polymerase (PARP), caspase-9, JAK-2, STAT-3, and β-actin monoclonal antibodies were purchased from Cell Signaling Technology. Cell Counting Kit-8 (CCK-8), Annexin-V-FLUOS staining kit, caspase-3 activity assay kit, and BCA protein assay kit were acquired from the Beyotime Institute of Biotechnology (Shanghai, China).

### 4.2. Preparation and Characterization of Naringenin

Naringenin was purchased from Shanghai Yuanye Biotechnology Co., LTD. The concentration of Naringenin was measured using inductively coupled plasma atomic emission spectroscopy. Naringenin was characterized using transmission electron microscopy (TEM, H-7650; Hitachi, Tokyo, Japan). The Zetasizer Nano ZS (Malvern Instruments Limited, Malvern, UK) particle analyzer was used to determine particle zeta potential and size distribution.

### 4.3. Cell Culture and Viability Assay

The HepG2 and LO2 cells were cultured at 37 °C in a humidified incubator containing 5% CO_2_. Antibiotics, fetal bovine serum (10%), 100 units of penicillin per ml, and 50 units of streptomycin per mL were added to Dulbecco’s Modified Eagle Medium [36]. As mentioned earlier, the inhibitory effect of different concentrations of naringenin on cell proliferation was measured using Cell Counting Kit-8. A 96-well plate was used for detection. Cells were added to each well at a density of 8000 cells/well, and the cells were maintained in 100% DMEM containing 10% FBS, in which the circle wells around the cells were blocked with 100 uL of PBS. Finally, the 96-well plate was placed in a 37 °C incubator for culturing. After the cells were fixed, there were six wells in each group. One group was the control group, and the other four groups were treated with different concentrations of naringenin (80 μM, 160 μM, 240 μM, and 360 μM) at 37 °C for 24 h. Cell medium with CCK-8 (10%; 100 uL) was added to each well and then incubated for 25 min at 37 °C in 5% CO_2_ [37]. After this, the color intensity of the formalin solution was measured at 450 nm using a microplate spectrophotometer (SpectroAmaxTM 250). An analysis of cell survival was conducted by comparing the absorbance percentages of treated cells [38].

### 4.4. Detection of Mitochondrial Membrane Potential (ΔΨm)

JC-1 fluorescent probe can detect the change of mitochondrial membrane potential (ΔΨm). Changes in mitochondrial function in HepG2 cells can be assessed with JC-1 assay. Cells were seeded into 6-well plates at a density of 1.6 × 10^5^ cells/well. After the cells adhered, different concentrations of naringenin were mixed in DMEM containing 10% FBS and treated for 24 h to observe the cell growth. First, the growth medium was removed, and cells were quickly washed with PBS. A total of 2 mL of JC-1 staining working solution was added to each well. After this, HepG2 cells were cultured at 37 °C for 30 min. Then, the supernatant was removed, and the cells were washed twice with 1 mL of frozen JC-1 staining buffer. Finally, 1 mL of culture medium was added to each well, and JC-1 fluorescence was detected using immunofluorescence microscopy. JC-1 fluorescence was measured with excitation (485 nm) and dual emissions (shift from green at 530 nm to red at 590 nm) [39].

### 4.5. Annexin-V/PI Double-Staining Assay

As mentioned earlier, HepG2 cells were stained with Annexin V-fluorescein isothiocyanate and PI according to the protocol included in the Annexin V-PI kit [40]. In brief, cells were treated with naringenin for 24 h. After the cells were washed with PBS, they were collected in a centrifuge tube and centrifuged at 1500 rpm for 5 min, and the original culture medium was removed. Next, the Annexin V-FITC binding solution, Annexin V-FITC, and PI were added and mixed well, and then stored at room temperature in the dark for 20 min. Finally, the cells were analyzed using flow cytometry. The condition of cell death was detected using a flow cellometer (Ex = 488 nm; Em = 530 nm). In addition, after the adherent cells were stained with Annexin-V/PI in a 6-well plate, they were observed in situ under a fluorescence microscope [41].

### 4.6. Cell-Cycle Analysis

The effect of naringenin on cell cycle distribution was detected using flow cytometry. [42]. Naringenin-treated cells were collected via centrifugation and fixed in ethanol overnight at 4 °C. The cells were then stained with PI dye for 30 min. DNA histograms were used to represent the proportion of cells in G0 versus G1, S versus G2, and the M phases, with the sub-G1 peak indicating the proportion of apoptotic cells [43]. The intracellular DNA content was detected using flow cytometry.

### 4.7. Caspase-3 and Caspase-9 Activity

Caspase-3 and caspase-9 activity assay kits were used to detect the activities of caspase-3 and caspase-9 in cells, respectively. In brief, HepG2 cells were treated with naringenin for 24 h, after which the medium was discarded [44]. Adherent cells were digested using trypsin, resuspended in a spare medium, and centrifuged at 1500 rpm for 5 min. The supernatants were then discarded, and the cells were washed once with PBS. Then, the cell lysates were added and cleaved for 15 min [45], before being centrifuged at 16,000× *g* for 15 min. After centrifugation, protein lysates were mixed with the reaction buffer (Ac-DEVD-pNA for caspase-3, Ac-LEHD-pNA for caspase-9) and incubated at 37 °C for 24 h in the dark. Enzyme activity was determined by measuring absorbance at 405 nm [41].

### 4.8. Determination of Reactive Oxygen Species

Mitochondrial dysfunction occurs when excessive ROS destroy mitochondria, degrade mitochondrial membrane potential (MMP), impair ATP production, and ultimately destroy the mitochondria [46]. ROS plays a pivotal role in the physiological process of apoptosis [47]. According to the previous experimental steps, the cells were inoculated on a 6-well plate and detected after naringenin treatment. The level of intracellular ROS can be measured using the fluorescence intensity of DCF [48]. At first, DCFH-DA was diluted with a serum-free medium at 1:1000 to a final concentration of 10 μM. Following treatment with naringenin, the cells were washed once with PBS. After PBS was removed, an appropriate amount of DCFH-DA diluent was added. Then, the cells were incubated at 37 °C for 20 min. The cells were washed with a serum-free medium three times to remove the DCFH-DA that had not completely entered the cells, and this whole process took place in a dark environment. The level of mitochondrial ROS was detected using 488 nm excitation and 525 nm emission, and the fluorescence intensity of DCF was analyzed. In addition, the adherent cells treated with naringenin for 24 h were directly incubated with diluted DCFH-DA at 37 °C for 20 min, washed three times with serum-free DMEM, and then directly observed using a laser confocal microscope [49].

### 4.9. Western Blot Analysis

Western blot was used to detect the expression of various intracellular proteins in naringenin-treated HepG2 cells. In brief, RIPA lysate was added to HepG2 cells to obtain total cellular proteins. Subsequently, the proteins were separated and transferred to cellulose acetate membranes according to previous methods. After incubation of PEG membranes with the first antibody and the appropriate second antibody, protein expression levels were measured using ECL chemiluminescence solution [50].

### 4.10. Statistical Analysis

All data are reported as mean ± SD. Multiple group comparisons were performed using a one-way analysis of variance (ANOVA). Differences of *p* < 0.05 (*), *p* < 0.01 (**), *p* < 0.001 (***), or *p* < 0.0001 (****) were considered statistically significant.

## 5. Perspectives and Conclusions

This study explored the effect of the natural product of naringenin on the apoptosis of HepG2 hepatoma cells, as well as the molecular mechanism by which naringenin inhibits the proliferation of HepG2 hepatoma cells at a cellular level. The main conclusions are as follows: By triggering apoptosis, naringenin can stop HepG2 hepatoma cells from proliferating. After being treated with naringenin, the cells had a rounded shape, their cell membrane permeability increased, their nuclei underwent pyknosis, their DNA seemed fragmented, and they displayed the typical signs of apoptosis. Naringenin decreased the potential of the mitochondrial membrane, which caused the formation of intracellular ROS. ROS not only stimulate caspase-9 and further activate caspase-3, which leads to the fragmentation of PARP, but also decrease the expression of the JAK-2 protein and STAT-3 protein. Finally, ROS induce the endogenous apoptosis pathway regulated by mitochondria and inhibit the growth and survival of tumor cells.

Naringenin is involved in different diseases (virus infection, Parkinson’s disease, cancer, diabetes, cardiac hypertrophy) and is becoming a key point for the individuation of a novel therapeutic target. According to the vitro experiment, naringenin, an effective inhibitor of HepG2 cells, could be an up-to-date approach. A further step will be required to elucidate the potential of naringenin in animal and clinical practice. This will be useful to develop effective drugs to treat HCC patients.

## Data Availability

Data sharing does not apply to this work, as no datasets were generated or analyzed in this study.
